# Oligomers of hepatitis A virus (HAV) capsid protein VP1 generated in a heterologous expression system

**DOI:** 10.1186/s12934-022-01780-x

**Published:** 2022-04-07

**Authors:** Anshu Nain, Mohit Kumar, Manidipa Banerjee

**Affiliations:** Kusuma School of Biological Sciences, Indian Institute of Technology Delhi, Hauz Khas, New Delhi, 110016 India

**Keywords:** Hepatitis A virus, VP1, Chaperone, GroEL, Refolding, Oligomers, Electron microscopy

## Abstract

**Background:**

The quasi-enveloped picornavirus, Hepatitis A Virus (HAV), causes acute hepatitis in humans and infects approximately 1.5 million individuals a year, which does not include the asymptomatically infected population. Several severe outbreaks in developing nations in recent years have highlighted the reduction in HAV endemicity, which increases the risk of infections in the vulnerable population. The current HAV vaccines are based on growing wildtype or attenuated virus in cell culture, which raises the cost of production. For generation of cheaper, subunit vaccines or strategies for antibody-based diagnostics, production of viral structural proteins in recombinant form in easily accessible expression systems is a priority.

**Results:**

We attempted several strategies for recombinant production of one of the major capsid proteins VP1, from HAV, in the *E. coli* expression system. Several efforts resulted in the formation of soluble aggregates or tight association of VP1 with the bacterial chaperone GroEL. Correctly folded VP1 was eventually generated in a discrete oligomeric form upon purification of the protein from inclusion bodies and refolding. The oligomers resemble oligomers of capsid proteins from other picornaviruses and appear to have the correct secondary and antigenic surface structure.

**Conclusions:**

VP1 oligomers generated in the bacterial expression system can be utilized for understanding the molecular pathway of HAV capsid assembly and may also have potential biomedical usages in prevention and diagnostics of HAV infections.

**Supplementary Information:**

The online version contains supplementary material available at 10.1186/s12934-022-01780-x.

## Background

Capsid proteins of mammalian viruses are often expressed in heterologous systems for vaccination, diagnostics and biotherapeutic delivery; as well as to study the pathways of capsid assembly at the molecular level. The yeast and insect cell based expression of the L1 protein of the Human Papilloma Virus (HPV) for vaccination against cervical cancer [1, 2], adeno-associated virus capsid protein VP3 expression in *E.coli* for facilitation of gene delivery [3], and bacterial expression of Cowpea Chlorotic Mottle Virus (CCMV) capsid protein for understanding the molecular mechanism of particle assembly [4] are a few instances of the versatile applications of virus structural proteins expressed in non-native conditions. Upon heterologous expression, viral capsid proteins frequently form higher order assemblies, including empty or partially filled virus-like particles that encapsulate non-native genomic material from the expression system [1, 2]. These assemblies not only provide information about the route and requirements for virus assembly, but also contain multivalent displays of antigenic sites, which can be an essential benefit for development of diagnostics or vaccine candidates [5].

Hepatitis A Virus (HAV), the causative agent of acute hepatitis in humans, is an unusual picornavirus which occupies the space between enveloped and non-enveloped viruses [6]. The capsid of HAV, like other picornaviruses, is formed from 60 copies of four capsid proteins—VP1, VP2, VP3 and VP4 [7]. A maturation cleavage event after capsid assembly and RNA encapsulation results in proteolytic cleavage of the precursor protein VP0 into VP2 and VP4. Capsid proteins VP1–VP3 are similar in size and display an eight-stranded β-barrel fold common to picornaviral capsid proteins. While the outer and inner capsid surfaces are constructed from these three proteins, the VP4 component, which is a small peptide, remains associated with the inner surface of the capsid [7]. This component is thought to be involved in cellular membrane penetration during HAV entry [8, 9]. The form of HAV circulating during infections contains a lipid coat (eHAV), although this form lacks embedded glycoproteins as found in traditionally enveloped viruses [6]. Partially due to this anomaly, the mechanistic details of HAV entry within host cells remains unclear. It is thought that the envelope is shed during the interaction of the virus particles with host cells, as the receptor binding sites are likely a part of the major capsid proteins.

The high-resolution structure of HAV displays closer similarity with picorna-like insect viruses rather than with authentic picornaviruses like Poliovirus or Foot-and-Mouth-Disease Virus (FMDV) [6]. The HAV capsid surface is neither as smooth as the FMDV capsid, nor does it contain the prominent depressions (canyons) at the bases of the pentamers as seen in enteroviruses like Poliovirus, that function as receptor-binding sites. In the absence of the canyons, it has been impossible to pinpoint potential receptor-binding sites on the HAV capsid. The identity of the receptor has not been entirely resolved as well. While the immunoglobulin-like protein HAVCR-1/TIM-1 was earlier thought to be the cellular receptor for HAV [10, 11], it has recently been found that HAV can invade cells lacking TIM-1 [12]. It has been proposed that gangliosides could be one of the key molecules promoting HAV entry as they allow lysosomal escape of HAV particles [12]. A recent cryo-EM structure of HAV bound to the potent R10 neutralizing antibody, which competes with the viral receptor for binding to the capsid surface, has indicated that the receptor probably binds to positively charged patches at the base of the pentamers [13]. The binding site for R10 is primarily localized to the VP3 capsid protein, however earlier studies with HAV immune escape variants have indicated that portions of VP1 constitute major antigenic determinants on the HAV capsid surface [13].

The immunodominant role of VP1 has been highlighted by the generation of virus-neutralizing antibodies produced against a synthetic peptide corresponding to the amino acids 11–25 of VP1 [14]. This region was selected based on surface similarities with a poliovirus neutralization site. Immune escape variants of HAV were found to have mutations at residues 102, 171, 176 and 221 of VP1 and residues 70, 71, 74 and 102–121 of VP3 [15]. It appears that the neutralizing antibody-binding sites for HAV encompass regions of VP3 and VP1 and may also contain stretches with amino acid contributions from both proteins [15–17]. Efforts have been made to recombinantly express the VP1 protein of HAV in *E.coli*, plant cells, insect as well as Drosophila cells [18–22]. A recent effort has shown the potential of utilizing recombinant VP1 as an effective reagent for diagnosis of HAV infections [22], instead of whole virus particles, which require a significantly larger investment of resources. The available vaccines against HAV are also based on infectious virus production, and the unusually long replication time of HAV in cell culture (2–3 weeks) compared to cytopathic viruses makes this process commercially and technically challenging [23].

We attempted to express the VP1 protein from the HM-175 strain of HAV in a bacterial system. Soluble protein, expressed with a GST tag, was found to associate tightly with the bacterial chaperone GroEL and could not be separated from the complex upon multiple attempts. A histidine tagged version of VP1 was recovered from inclusion bodies and refolded. The refolded protein assembled into multimeric forms with structural similarities to dissociated picornavirus capsid proteins and cross-reacted against an anti-HAV polyclonal antibody. The multimers of HAV could be utilized in future for a molecular level understanding of HAV assembly and could also find usage in diagnostics and disease prevention.

## Results

### Expression of VP1 in soluble form in *E. coli*

**Fig. 1:**
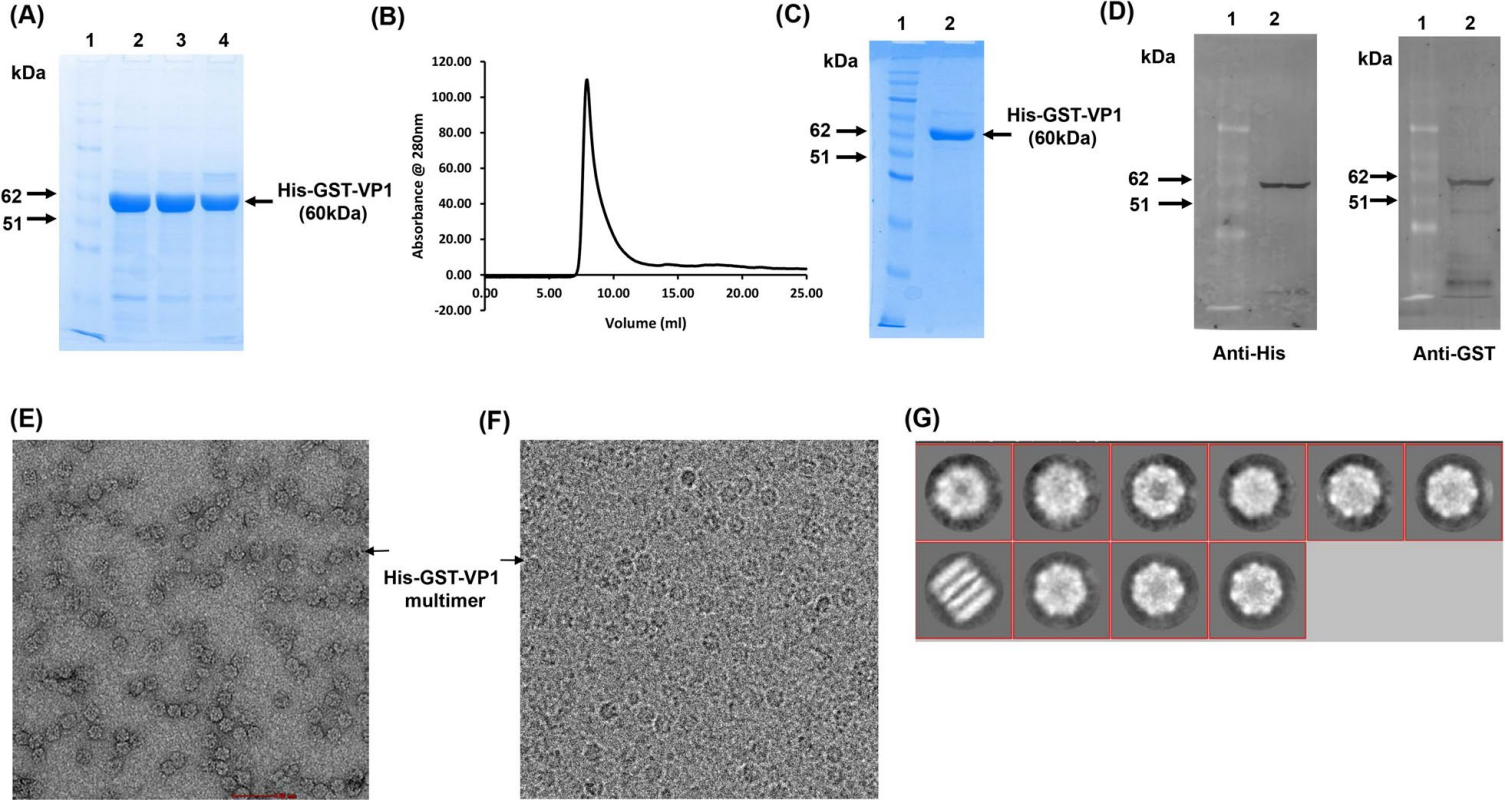
Expression and Purification of His-GST-VP1 from *E.coli*, **A** His-GST-VP1 purified using nickel chelation chromatography analyzed on 8% SDS-PAGE. Lane 1 represents the protein markers, and lanes 2–4 represent protein fractions eluted with 350 mM imidazole. **B** Elution profile of His-GST-VP1 from a Superdex 200 (10/300) size exclusion column. **C** Purified protein from size exclusion chromatography analyzed on 10% SDS-PAGE. Lane 1 represents the protein markers, and lane 2 represents the peak fraction. **D** Western blots showing the cross reactivity of the purified protein with anti-His (left) and anti-GST (right) antibodies. **E** and **F** shows His-GST-VP1 multimers visualized by negative stain and cryo transmission electron microscopy respectively. **G** shows a 2D class average of His-GST-VP1 multimers from cryo-micrographs

HAV VP1 was tagged with an N-terminal Glutathione S-transferase (GST) to support soluble protein expression in the bacterial system. A 6X-Histidine tag was also added to the construct for ease of purification. Purification using Ni-NTA chromatography (Fig. 1A), followed by size exclusion chromatography, resulted in the protein being eluted in the void volume, indicating formation of large aggregates (Fig. 1B–D). Cryoelectron microscopy and image reconstruction showed the presence of sevenfold symmetry indicated the involvement of the bacterial chaperone GroEL (Fig. 1E–G). LC–MS/MS based analysis of the 60 kD band corresponding to purified His-GST-VP1 on SDS-PAGE showed the presence of VP1, GST as well as GroEL (Additional file 1: Fig. S1A and B), indicating close association of the tagged VP1 with the chaperone, which also has a dissociated mass of 60 kD. Since strong association of GST with GroEL was previously reported [24], we attempted to express VP1 with only a His-tag in Rosetta cells. However, His-VP1 also appeared to form a strong binary complex with GroEL (Additional file 2: Fig. S2A–C). Several unsuccessful attempts were made to separate VP1 from GroEL by using either ammonium sulfate precipitation or an ATP gradient (data not shown). Expression of His-tagged VP1 in *E.coli* ArcticExpress (DE3) competent cells, which constitutively express the cold-adapted Cpn10 and Cpn60 chaperonins from the psychrophilic bacterium *O. Antarctica*, was attempted at lower temperature to ensure correct folding; however this also resulted in strong association of VP1 with the Cpn60/Cpn10 complex (Additional file 3: Fig. S3A). Further, expression of His-VP1 in a chaperone deficient *E.coli* strain BB1553 resulted in purification of VP1 (Additional file 3: Fig. S3B and C), although it was in the form of large soluble aggregates, as indicated by Dynamic Light Scattering (DLS) and transmission electron microscopy (Additional file 4: Fig. S4A and B respectively).

### Purification of VP1 in denatured form and refolding

**Fig. 2:**
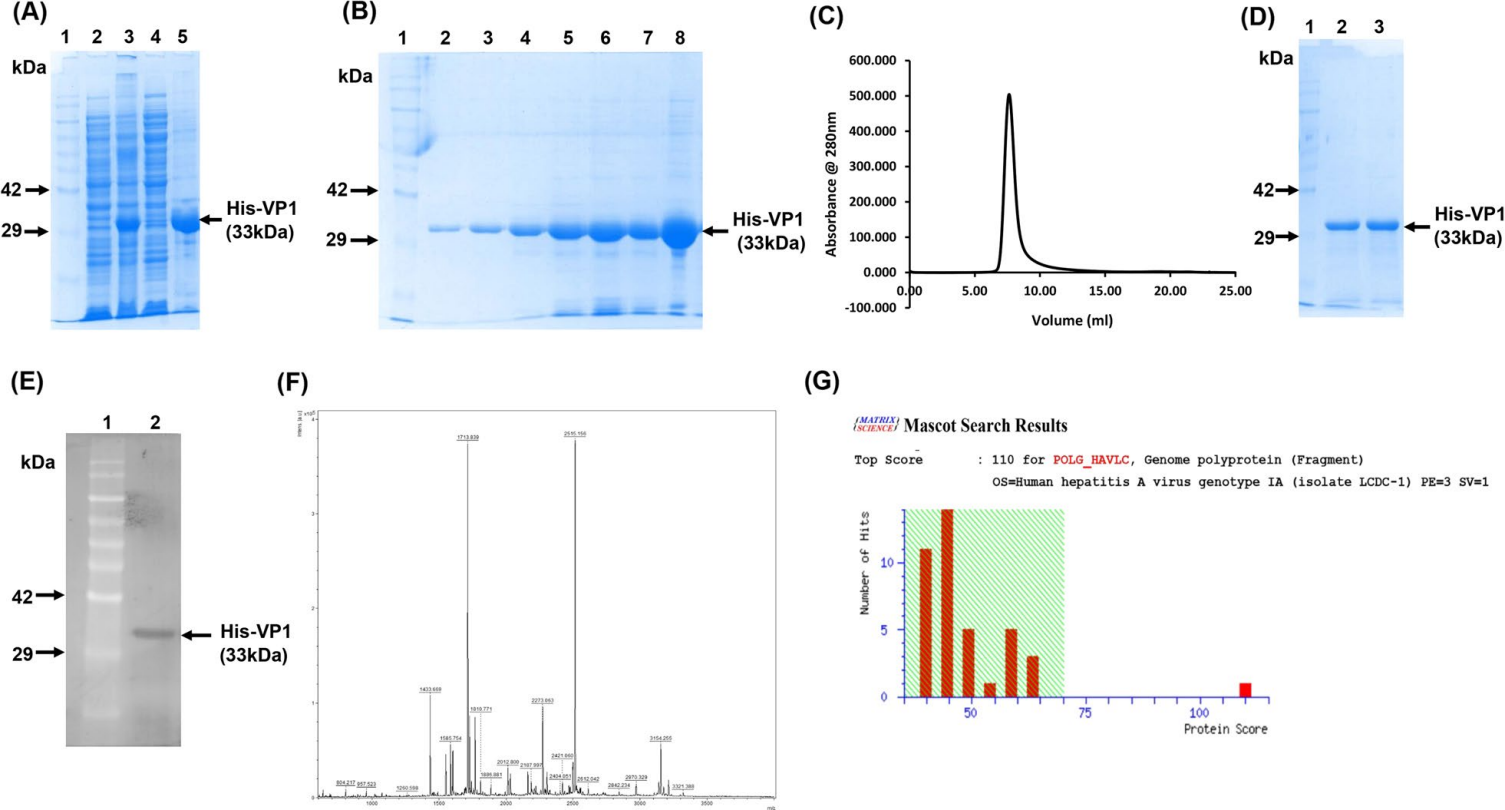
Expression and purification of His-VP1 under denaturing conditions. **A** Soluble and pellet fractions of bacterial cells expressing His-VP1 analyzed on 10% SDS-PAGE. Lane 1 represents the protein markers, lane 2 and 3 represents the cellular protein fractions before and after IPTG induction, lane 4 represents the supernatant containing *E.coli* soluble proteins, and lane 5 represents the insoluble fraction containing His-VP1 **B** His-VP1 purified using nickel chelation chromatography under denaturing conditions analyzed on 10% SDS-PAGE. Lane 1 represents the protein markers, and lanes 2–8 represent protein fractions eluted with 350 mM imidazole. **C** Elution profile of His-GST-VP1 from a Superdex 200 (10/300) size exclusion column. **D** Purified protein from size exclusion chromatography analyzed on 10% SDS-PAGE. Lane 1 represents the protein markers, and lanes 2 and 3 represent the peak fraction. **E** Western blot showing the cross reactivity of the purified protein against an anti-HAV polyclonal antibody. **F** LC–MS/MS analysis showing mass spectra of trypsin digested VP1. **G** Peptide mass fingerprinting and database search showing the presence of HAV VP1

To prevent misfolding, formation of soluble aggregates and robust association with bacterial chaperones, we attempted to purify HAV VP1 as inclusion bodies, followed by refolding. Overexpression of the His-VP1 construct was carried out in BL21 (DE3) cells at 37 °C with 1 mM IPTG for 4 h, and His-VP1 produced as inclusion bodies (Fig. 2A) was solubilized with 8 M urea. The denatured protein was purified by Ni-NTA chromatography (Fig. 2B) and refolded by step wise dialysis in phosphate buffered saline with gradually decreasing concentration of urea. Post dialysis, the refolded protein was subjected to size exclusion chromatography for further purification and also to analyze the oligomeric state of the protein. A monodisperse peak was obtained (Fig. 2C), which was collected and analyzed on 10% SDS-PAGE (Fig. 2D). Western blotting with an anti-HAV polyclonal antibody (Fig. 2E) and mass spectrometric analysis (Fig. 2F) was carried out to confirm the identity of the purified HAV VP1. The peptide spectra (Fig. 2G), when searched against the Swiss-Prot database, matched to VP1 from HAV with a significant mascot score (110). The yield of the refolded protein was 8 mg per liter of bacterial culture.

### Secondary structure and oligomeric state of refolded HAV VP1

**Fig. 3:**
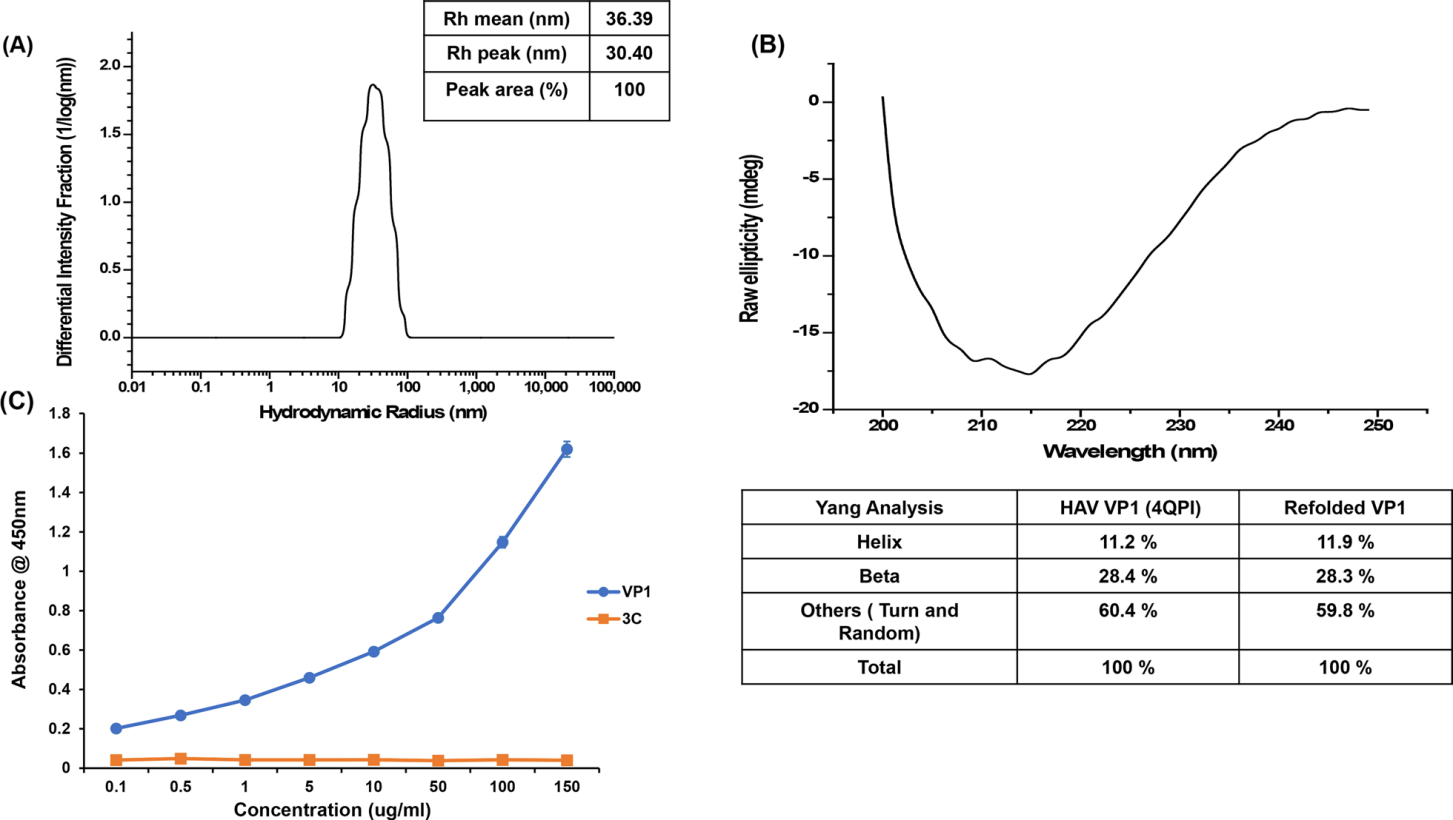
Size distribution, secondary structure and antigenicity of refolded VP1. **A** Dynamic Light Scattering (DLS) of refolded VP1, with the Rh_mean_, Rh_peak_ and peak area percentage in the inset table. **B** Circular dichroism (CD) spectra of refolded VP1 with a table showing the percentage of secondary structure components as calculated by the Spectra Manager software of JASCO. **C** The cross reactivity of refolded VP1 (blue) and recombinantly generated HAV non-structural protein 3C^pro^ (orange) against a polyclonal anti-HAV antibody analyzed by ELISA. The graph represents the average OD_450_ for three different concentrations of VP1 and 3C^pro^. Values are represented as mean ± SD

We found that the refolded protein eluted in the void volume of the Superdex 200 column, indicating that it is not in a monomeric state. However, Dynamic Light Scattering (DLS) of refolded HAV VP1 uniformly showed a monodisperse peak upon multiple attempts (Fig. 3A), indicating formation of a consistent higher order structure. The mean hydrodynamic radii (Rh_mean_) of the refolded protein was ~ 36 nm (Fig. 3A, inset), which was substantially different from the Rh_mean_ of ~ 900 nm obtained for soluble aggregates (Additional file 4: Fig. S4A). This signifies that the refolded protein is structurally distinct from soluble, aggregated species. To analyze whether the refolding of the protein is accurate, we carried out circular dichroism (CD) spectroscopy. The CD spectra of the refolded VP1 protein indicated an α-helical content of 11.9%, a β-sheet content of 28.3%, the rest being turns and coils (Fig. 3B). This secondary structural content closely matched that of the VP1 component from the X-ray crystallographic structure of the HAV capsid, which displays an eight-stranded β-barrel structure. Thus, we concluded that the refolded HAV VP1 is likely correctly folded, and the elution of the protein in the void volume during size exclusion chromatography likely indicates the formation of higher order structures, rather than random, large aggregates.

### Antigenicity of refolded HAV VP1

To further validate that refolded HAV VP1 is in a correctly folded form, its ability to bind to a polyclonal antibody against the HAV capsid was determined using an ELISA assay. Recombinant, His-tagged, 3C protease, a non-structural component of the HAV capsid generated in *E.coli* [25] was utilized as a negative control. While the refolded His-tagged VP1showed appreciable binding to the antibody, there was no cross-reactivity against HAV 3C (Fig. 3C). This showed that the refolded HAV VP1 has exposed antigenic surfaces similar to that present in the native virus, indicating the accuracy of the refolding.

### Structural analysis of refolded HAV VP1

**Fig. 4:**
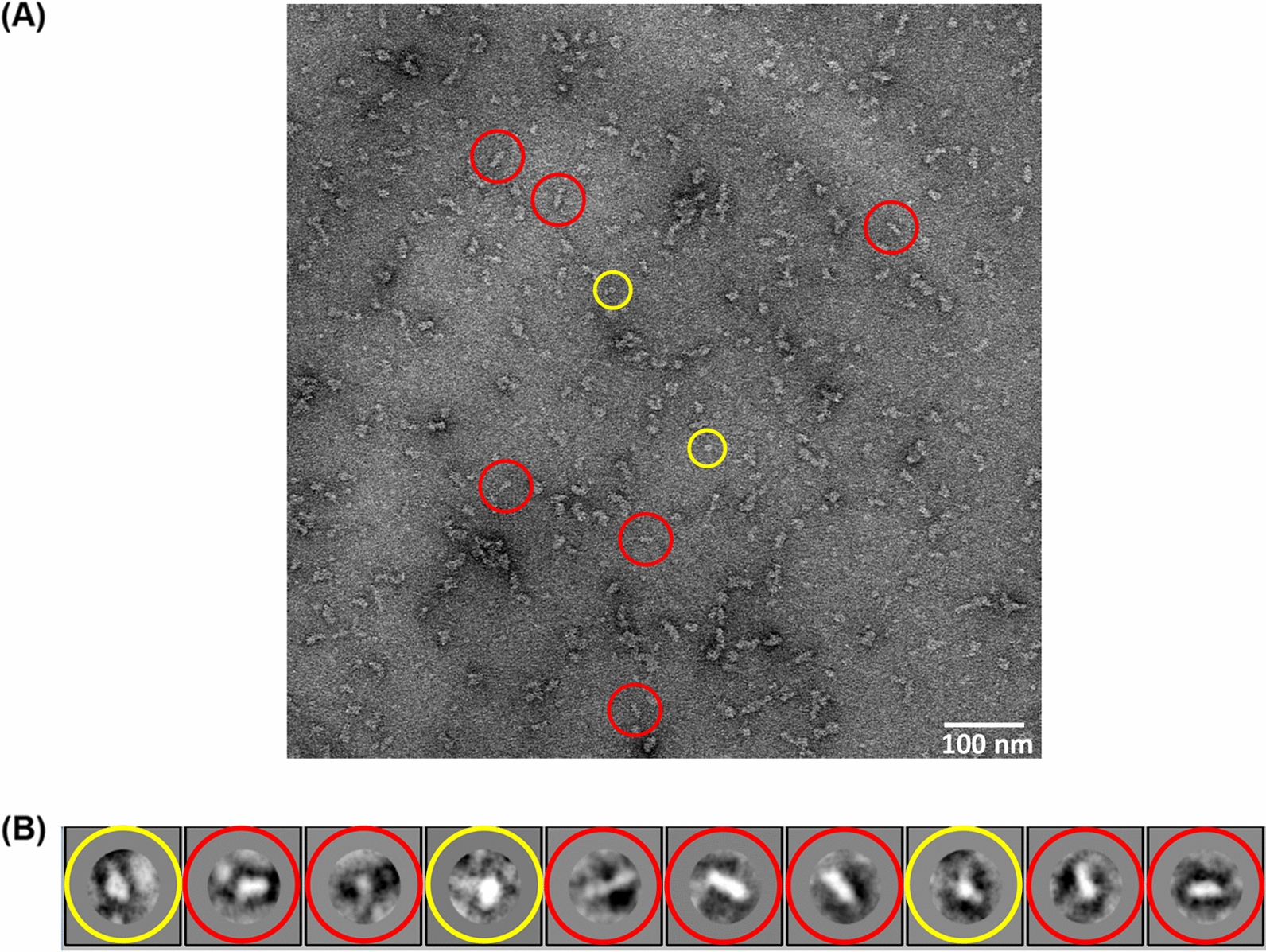
Morphological analysis of refolded VP1 using electron microscopy. **A** A micrograph of negatively stained refolded VP1, showing the top and side orientations highlighted in yellow and red circles respectively. (Scale bar 100 nm, Magnification 50k×). **B** 2D classification of refolded VP1 showing 10 different classes with both top (yellow) and side (red) views

The refolded protein was visualized using negative stain electron microscopy, which showed a distribution of particles with structural uniformity (Fig. 4A). It is possible that the particles denote formation of multimeric complexes of VP1, and show similarities with the pentameric complexes of capsid proteins generated during disassembly of Foot-and-Mouth-Disease-Virus (FMDV) capsids [26]. A close look at the particles indicated the presence of different orientations—including images representing possible side views (highlighted in red) and top or bottom views (highlighted in yellow) (Fig. 4A). The latter were similar to the top views of FMDV VP1 pentamers [26]. 458 particles were manually picked from 22 micrographs and subjected to 2D classification. Ten discrete classes with representations from both top and side views were obtained (Fig. 4B).

**Fig. 5:**
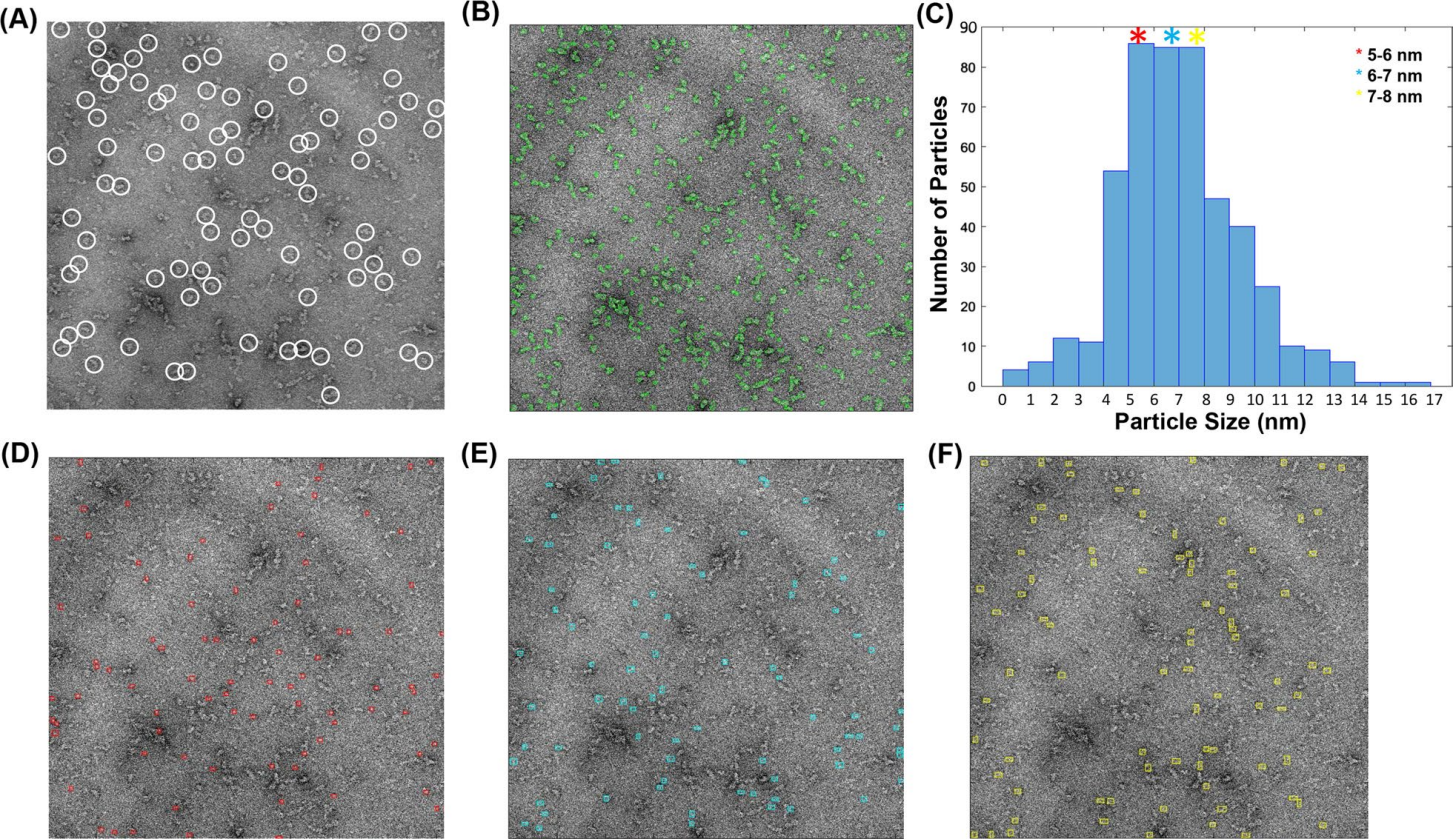
Semi-quantitative analysis of refolded VP1 using SoftEM. **A** Manually picked particles for 2D classification, **B** particles picked using SoftEM, **C** size distribution of particles based on their equivalent radii in nm. **D**–**F** show particles with equivalent radii within 5–6 nm, 6–7 nm, and 7–8 nm picked in red, cyan and yellow respectively, which have been quantified in **C**

In order to remove any bias from manual particle picking, a homegrown software, SoftEM was utilized for automated particle picking (Fig. 5A–F). SoftEM denoises electron micrographs by flattening noisy backgrounds, followed by Otsu thresholding and identification of particles by a connected component labelling method in a semi-automated fashion. Numerical analysis and size distribution of particles is carried out based on their equivalent radii in nm [27]. For the micrographs of refolded His-VP1, the majority of the particles picked were found to have diameters between 5 and 8 nm (Fig. 5C). The size distribution bar showed three different peaks with the maximum number of particles, with diameters ranging between 5 and 8 nm using selective labelling (Fig. 5C). Selective labelling enables a user to pick same sized complexes based on their equivalent radii from a pool of picked particles [27] (Fig. 5D–F). Particles were also picked manually for comparative purposes and showed good agreement with semi-automated picking.

The distinction between the particle sizes obtained by DLS and TEM is probably because while the former measures the hydration sphere diameter of a population which is influenced by the presence of small quantities of larger aggregates, the latter method carries out a number-weighted size distribution of dehydrated particles. In the latter case, the presence of small quantities of larger aggregates is not expected to influence the quantification of particle size. Further, the TEM based quantification method calculates equivalent radii of particles, which may not represent the exact dimensions of the particles. The presence of some larger aggregates in the sample is supported by the broad distribution of the DLS peak (10–100 nm). It is possible that some of the larger aggregates could have been disproportionately removed during TEM because of multiple factors, including lack of association of the larger species with the grid, loss of aggregates during washing steps, or lack of visualization at higher magnifications.

## Discussion

Hepatitis A Virus is the causative agent of acute viral hepatitis, which is usually self-limiting, but can occasionally turn into fulminant hepatitis or liver failure [23, 28, 29]. HAV-mediated hepatitis, in most South Asian countries, was initially an asymptomatic childhood disease, however, a fast improvement in socio-economic conditions and other factors over the last two decades has shifted the epidemiology of the disease to symptomatic infections in teenagers and young adults, and increased the chances of large-scale outbreaks, as in Eastern European countries and China over the last two decades [30, 31]. Various national and international agencies have suggested routine childhood immunizations against HAV, particularly in countries with low and intermediate endemicity to prevent future outbreaks [31, 32]. As of 2019, 34 countries had included or were planning to include HAV vaccines in routine childhood immunizations. However, the prohibitive cost of available vaccines make these efforts untenable in third world countries like India [23, 31]. Although several versions of inactivated viral vaccines, like Havrix or Twinrix are available, they are based on initially generating the virus in culture [30]. HAV is very slow growing in culture and procuring large quantities of purified virus drives up the cost of generating inactivated or live attenuated vaccines. Under the given circumstances, generating cheaper subunit vaccines against HAV is a priority. Recombinantly generated HAV structural proteins may also be utilized for other usages, like development of effective diagnostic tests.

Due to their potential usage in diagnostics and vaccine candidates, many efforts have been made to generate the structural proteins of HAV in a variety of heterologous systems [18, 19, 22]. Expression of mammalian virus proteins in the bacterial system is challenging due to the problems associated with rare codons, lack of post-translational modification, and abundance of proteases in *E.coli*. Protein misfolding, formation of inclusion bodies, and proteolytic degradation are common problems associated with of recombinant expression in *E.coli*. In spite of these problems, *E.coli* is still a preferred expression host due to its robustness, ease of manipulation, time and cost effectiveness and high yields compared to other expression systems like insect or mammalian cells. We chose to express VP1 in the bacterial system in conjunction with a Glutathione-S-transferase (GST) tag. It is well documented in literature that the fusion of highly soluble GST to partners prone to misfolding or aggregation is an efficient and effective approach for the production of soluble and correctly folded protein. VP1 is hydrophobic in nature and there could be a high possibility of its misfolding or aggregation when expressed in *E.coli*, which could be potentially overcome by the inclusion of GST. However, we found that our His-GST-VP1 fusion protein was tightly associated with the bacterial chaperone GroEL, and co-eluted with the chaperone upon size-exclusion chromatography. We confirmed that the association is primarily due to the VP1 component, however, implementation of multiple strategies such as protein expression at reduced temperature, inclusion of detergents and chaotropes, expression in ArcticExpress cells, separation by ammonium sulphate precipitation or ATP gradient, were all unsuccessful in separating recombinant VP1 from bacterial chaperones. It has recently been shown that too tight of an association between the client protein and chaperone may slow protein folding drastically, and a moderate level of binding is required to obtain optimal results [33]. It is possible that the association between HAV VP1 and GroEL is unusually tight, which hinders correct folding and release of VP1 from the chaperone. Attempts to decipher the structural basis of the interaction of GroEL with the recombinant protein was unsuccessful due to the current low resolution of asymmetric 3D reconstruction of the His-GST-VP1-GroEL complex. Although a reconstruction carried out by imposing sevenfold symmetry converged at a resolution of 13 Å, the density for His-GST-VP1 was not visible inside the GroEL cage, which is likely due to the asymmetric nature of the interaction between GroEL and VP1. A high-resolution symmetry-free reconstruction will likely be required for a molecular level explanation of the association of VP1 with the chaperone. Efforts to express His-VP1 in a chaperone deficient cell line resulted in formation of large soluble aggregates and was not pursued further.

Typically, production of recombinant proteins in the soluble form is preferred due to ease of large-scale/industry level scale-up. However, due to the problems associated with soluble expression, we attempted to refold HAV VP1 after purification from inclusion bodies under denaturing conditions. Stepwise refolding by dialysis resulted in the production of recombinant VP1 which appeared to have the correct secondary structure profile and antigenicity, however, was still found to elute as a large molecular weight species upon size exclusion chromatography. Visualization by electron microscopy established the formation of oligomeric forms of VP1. The orientations presented by the particles appear to have notable structural similarities with the pentamers of capsid proteins generated during disassembly of the FMDV capsid [26]. Manual as well as automated particle picking methods identified the oligomeric structures from negatively stained micrographs and a 2D classification indicated the presence of side and top/bottom orientations. However, it is to be noted that unlike the pentamers generated during FMDV disassembly, which contain the capsid proteins VP1, VP2 and VP3, the oligomeric forms reported by us are generated from recombinant VP1 only. The mode of oligomerization of recombinant HAV VP1 may be different from that of picornaviral capsid proteins during native virus assembly. There could also be heterogeneity in the oligomeric forms generated by HAV VP1, and a cryoelectron microscopy based 3D reconstruction of the particles will be needed in order to address these questions.

It has been proposed that assembly of picornaviruses is initiated by the self-association of capsid protein VP1 into pentamers [34]. In case of HAV, the self-association is thought to be initiated by the VP1-2A unit, with the non-structural protein 2A being primarily responsible for virion assembly [35]. However, the molecular mechanism of the process is not clearly understood. Biophysical analysis of HAV assembly or disassembly have been largely precluded due to the difficulties associated with generating substantial quantities of HAV particles for such analysis [23]. Here, for the first time, we report oligomeric assembled units of HAV VP1 generated in a heterologous expression system. High resolution structural studies of these units will help in deciphering the early events of HAV assembly. Further, the antigenicity and mutimeric nature of the assembled particles also indicate their potential suitability as a cheaply produced, stable and effective subunit vaccine candidate against HAV.

## Conclusions

Recombinant production of viral capsid proteins is essential for development of diagnostics and vaccination strategies; however large-scale production in an accessible and cheap expression system presents several difficulties. Currently available HAV vaccines are based on whole virus production in cell culture and are consequently expensive. We present a method for production of one of the major antigenic determinants of the HAV capsid, the VP1 protein, from *E.coli*. Our data shows tight association of VP1 with the bacterial chaperone GroEL, which hindered correct folding, and was overcome by purification of the protein in denatured state and in vitro refolding. Our data suggests that recombinant VP1 can form oligomers in absence of the viral non-structural protein 2A and presents correct antigenic surfaces. Bacterially generated recombinant VP1 can be utilized to understand virus assembly, as well as for diagnostics and disease prevention.

## Materials and methods

### Cloning

The region corresponding to VP1 (819 bp), from the cDNA corresponding to the genome of HAV strain HM175/18f, was cloned in the bacterial expression vector pGEX-6P-2 between the BamHI and XhoI restriction sites. This resulted in the inclusion of an N-terminal GST tag. GST-VP1 was then amplified and cloned in the bacterial expression vector pET28b between the NheI and XhoI restriction sites, resulting in the inclusion of a 6X-His tag N-terminal to GST-VP1. For cloning of the His-VP1 construct, the cDNA corresponding to VP1 was amplified via PCR with forward (5′ AAAGCTAGCGTTGGAGATGATTCTGGAGG 3′) and reverse (5′ AAACTCGAGTCACTCAAATCTTTTATCTTCCTCTG 3′) primers using the plasmid pHM175/18f as a template and cloned in between NheI and XhoI restriction sites of pET28b. All constructs were confirmed by sequencing with T7 forward and reverse primers.

### Protein expression and purification

The GST-VP1 construct was transformed in competent *E.coli* strain Rosetta (DE3) pLysS cells. Cultures were grown in Luria Bertaini (LB) broth at 37 °C until the OD_600_ reached 0.6, when protein overexpression was induced by addition of 0.5 mM IPTG. Induction was carried out at 37 °C for 2 h in the presence of 50 mM proline to enhance protein solubility. Cells were harvested, resuspended in a lysis buffer containing 50 mM NaH_2_PO_4_ pH 8.0, 500 mM NaCl and 1 mM PMSF, and disrupted by lysozyme treatment, followed by sonication. His-GST-VP1 was purified by Ni-NTA affinity chromatography. After elution with 350 mM imidazole, the protein was dialyzed in a buffer containing 50 mM NaH_2_PO_4_ pH 8.0, 500 mM NaCl, 2 mM DTT and 2.5 mM EDTA, and loaded on a Superdex 200 column (10/300, GE Healthcare). Fractions containing the protein were collected and analyzed on 10% SDS-PAGE. Overexpression of His-VP1 in rare codon-optimized *E.coli* strain Rosetta (DE3) pLysS was induced for 18 h at 18 °C with 0.1 mM IPTG concentration, that in *E.coli* ArcticExpress (DE3) cells was carried out at 12 °C, with 1 mM IPTG for 24 h, while that in DnaK deficient strain BB1553 was carried out at 20 °C, with 1 mM IPTG for 20 h. Purification was carried out similarly by Ni-NTA affinity chromatography, followed by size-exclusion chromatography. To optimize correct folding and solubility of His-VP1, various media like 2XYT and additives like 10 mM proline, 0.5% CHAPS, and 10% glycerol were utilized.

### Purification from inclusion bodies and refolding

For purification from inclusion bodies, His-VP1-pET28b construct was transformed in *E.coli* strain BL21 (DE3), and protein overexpression was carried out at 37 °C for 4 h induced with 1 mM IPTG. Post induction, cells were harvested by centrifugation at 6000 rpm for 20 min at 4 °C and resuspended in 20 mM Tris–HCl pH 8.0. Cell lysis was carried out by sonication, and the resultant pellet was resuspended in a denaturing buffer containing 10 mM PBS, pH 7.4 and 8 M urea to solubilize the inclusion bodies. The urea-solubilized denatured protein was allowed to bind to Ni-NTA agarose beads, followed by washes in denaturing buffer containing 20 mM imidazole, and elution in the same buffer containing 350 mM imidazole.

The eluted protein was dialyzed against buffer solutions containing 10 mM PBS, pH 7.4 with gradually decreasing concentrations of urea, at 4 °C with gentle stirring. Post dialysis, the solution was centrifuged at 12,000*g* for 10 min at 4 °C to remove any precipitated protein. The cleared supernatant was loaded on a Superdex 200 column (10/300) fitted to an AKTA purifier FPLC system (GE Healthcare) equilibrated in the same buffer for further purification by size exclusion chromatography. Fractions were collected and analyzed on SDS-PAGE.

### Western blotting

For Western blotting, the protein sample separated on 10% SDS-PAGE was transferred to a PVDF membrane. The membrane was blocked with 5% skimmed milk in TBST, followed by incubation with an anti-HAV polyclonal primary antibody (Thermofisher Scientific), and a rabbit anti- goat secondary antibody conjugated with HRP (Thermofisher Scientific). All washes were carried out in TBST. The membrane was treated with an ECL Plus chemiluminescent substrate reagent kit (Thermo Scientific, USA) and developed on a Typhoon imager (Typhoon FLA 9000, GE).

### Mass spectrometry

For LC–MS/MS analysis, the band corresponding to the protein of interest was excised manually from a coomassie stained acrylamide gel, cut into small pieces, washed with acetonitrile: H_2_O (50%, v/v) containing 50 mM NH_4_HCO_3_ to remove the dye, and digested into smaller peptides using trypsin. An extraction buffer containing trifluoroacetic acid/acetonitrile (1:1, v/v) was added to the digested peptides, followed by sonication for 10–15 s, and incubation at 25 °C for 45 min. The extracted peptide mixture was desalted using C18 tips and analyzed by LC–MS/MS. The spectra were screened against Swissprot databases for peptide mass fingerprinting using the Mascot search engine (Matrix Sciences, UK) with the parameters (mass values = monoisotopic, mass tolerance ± 100 ppm and maximum missed cleavage = 1).

### Circular dichroism spectroscopy

Circular dichroism (CD) spectroscopy of purified VP1 was performed on a Peltier-attached JASCO J-815 spectropolarimeter (Tokyo, Japan). The purified protein at a concentration of 0.2 mg/ml in PBS was placed in a 1 mm path-length quartz cuvette and the spectra was measured in the far-UV region (200–250 nm). Spectra were taken in triplicates and then averaged. Secondary structures percentage analysis was done using JASCO’s Spectra Manager software.

### ELISA

The cross-reactivity of purified VP1 with an anti-HAV polyclonal antibody was measured via an indirect enzyme-linked immunosorbent assay (ELISA). Briefly, purified and refolded VP1 in 10 mM PBS buffer pH-7.4 at concentrations ranging from 0.1 to 150 μg/ml, was used to coat a 96-well, flat-bottom ELISA plate and incubated at 4 °C overnight. Control wells were set aside for the negative, blank and positive controls. HAV non-structural protein 3C, purified from *E.coli* [25] was used as a negative control while the inactivated viral vaccine HAVRIX vaccine was used as a positive control. The plates were incubated with an anti-HAV polyclonal primary antibody (PA1-73089 Thermofisher Scientific, generated against the HM175 strain of HAV) followed by a Horseradish peroxidase (HRP) conjugated rabbit anti-goat secondary antibody (Thermofisher Scientific) at 37 °C for 1 h. All washes were carried out in PBS containing 0.05% Tween (PBS-T). For colour development, 100 μl TMB substrate solution (BD) was added to each well, followed by incubation at room temperature for 20 min in the dark, and addition of a 100 μl stop solution (2 N H_2_SO_4_). Absorbance at 450 nm was measured using a ELISA plate reader (Bio-Rad).

### Dynamic light scattering

Dynamic light scattering (DLS) was carried out using a miniDAWN Tristar laser photometer with a Wyatt quasi-elastic light scattering (QELS) attachment (Wyatt Technology Corp., Santa Barbara, CA). The detector was equipped with a 682 nm laser and data was collected at 90° scattering angle. 50 µl of purified VP1 at a concentration of 0.2 mg/ml in 10 mM PBS, pH 7.4 was placed in a quartz cuvette for DLS, and parameters such as Rh_mean_, Rh_peak_ and peak area percentage were calculated using the inbuilt Astra software.

### Separation of VP1 from GroEL

Ammonium sulphate precipitation and ATP gradient methods were attempted to separate His-VP1 from GroEL. In the first case, samples containing His-VP1 and GroEL were treated with a gradient of 5–20% ammonium sulphate supplemented with 10% glycerol, and subjected to gentle shaking for 1 h at 4 °C. The sample was centrifuged at 12,900 g at 4 °C, and analyzed on SDS-PAGE. In the ATP gradient method, the Ni-NTA bound protein was washed with a gradient of 5, 10, 20 and 50 mM ATP along with 20 mM MgCl_2_, followed by elution with 350 mM imidazole. All samples were analyzed on 10% SDS-PAGE.

### Electron microscopy and reconstructions

For negative stain electron microscopy, 4 μl of sample was allowed to adsorb on a glow discharged 200-mesh, carbon coated copper grid for 1 min. The surplus sample was removed using a whatmann filter paper, followed by rinsing the grid three times with double distilled water (ddH_2_O). For staining, 4 µl of 2% uranyl acetate solution was applied onto the grid for 1 min. After removal of the excess stain, the grid was air-dried and visualized on a 200 kV FEI Tecnai™ transmission electron microscope (TEM) operating at 80kx magnification.

For cryoelectron microscopy, 3 µl of the His-GST-VP1-GroEL complex was adsorbed on a plasma cleaned, 300-mesh quantifoil holey carbon grid, followed by plunge freezing using a Vitrobot (Mark IV, Thermo Scientific). The parameters for plunge freezing were—temperature 11 °C, 100% humidity, blot time 3 s, blot force 1. The grids were stored in liquid nitrogen, and data was collected at the CBIS facility, National University of Singapore on a Titan Krios cryomicroscope (Thermo Scientific) equipped with a direct electron detector at a magnification of 71000x, and at a low electron dose of 6–10 electrons/Å2, with a defocus ranging from − 0.5 to − 3 µm. A total of 2300 micrographs were collected, which were processed using RELION [36]. 13,000 particles were used for 2D classification, which resulted in 10 different classes. The 6 best classes were used to generate the initial 3D model of the complex. The initial model of the complex converged at ~ 13 Å after 25 iterations, on applying C7 symmetry. A symmetry-free reconstruction converged at ~ 20 Å after 25 iterations.

2D classification of refolded His-VP1 particles was carried out from negatively stained particles using RELION. 22 negatively stained micrographs were pre-processed and contrast transfer function (CTF) corrected for manual particle picking. 458 particles were classified into 10 different classes. Seven classes showed elongated structures (side view) and 3 classes showed round structures (top view) of refolded His-VP1.

### Particle distribution analysis

Negatively stained micrographs of refolded His-VP1 particles were further processed by a homegrown software, SoftEM, for semi-quantitative characterization [27]. In addition to automated picking, particles were also picked manually for comparison. Automatically picked particles were subjected to quantitative distribution based on their equivalent radii.

## Supplementary Information

**Additional file 1: Figure S1.** Association of His-GST-VP1 with bacterial chaperone GroEL determined by LC–MS/MS **(A)** LC–MS/MS analysis showing mass spectra of trypsin digested, purified protein **(B)** Peptide mass fingerprinting followed by database search confirmed the presence of GroEL, VP1 and GST.

**Additional file 2: Figure S2.** Expression and Purification of His-VP1 from *E.coli* strain Rosetta (DE3) pLysS cells **(A)** Elution profile of His-VP1 from a Superdex 200 (10/300) size exclusion column **(B)** Purified protein from size exclusion chromatography analyzed on 10% SDS-PAGE, showing the presence of both GroEL and His-VP1 at 60 kDa and 33 kDa respectively. Lane 1 represents the protein markers, lane 2 represents Ni–NTA purified fraction prior to SEC, lanes 3 to 7 represent the peak protein fraction from SEC **(C)** A complex of HisVP1-GroEL visualized by transmission electron microscopy.

**Additional file 3: Figure S3.** Expression and purification of His-VP1 from ArcticExpress (DE3) and BB1553 *E.coli* strains **(A)** Purified His-VP1 from ArcticExpress (DE3) cells analyzed on 8% SDS-PAGE. Size exclusion chromatography resulted in co-elution of Cpn60 and His-VP1. Lane 1 represents the protein markers, while lanes 2 to 6 represent the peak fraction from SEC. **(B)** Expression and purification of His-VP1 from *E. coli* strain BB1553 deficient in DnaK bacterial chaperone**.** Ni–NTA purified protein fractions analyzed on 10% SDS-PAGE. Lane 1 represents the protein markers, and lanes 2 to 7 represent protein fractions eluted with 350 mM imidazole. **(C)** SEC purified fractions of His-VP1 from *E. coli* strain BB1553 analyzed on 10% SDS-PAGE. Lane 1 represents the protein markers, lane 2 represents Ni–NTA purified fraction prior to SEC, lanes 3 to 7 represent the peak protein fraction from SEC.

**Additional file 4: Figure S4.** Size distribution and morphological characterization of VP1 purified from *E. coli* strain BB1553. (A) Dynamic Light Scattering (DLS) of purified VP1, with the Rh_mean_, Rh_peak_ and peak area percentage in a tabular form. (B) Presence of protein aggregate visualized by transmission electron microscopy.

## Data Availability

All data generated or analysed during this study are included in this published article, and its Additional files. The materials are available from the corresponding author on reasonable request.

## Abbreviations

HAV: Hepatitis A virus
VP: Viral protein
HPV: Human Papilloma Virus
CCMV: Cowpea Chlorotic Mottle Virus
FMDV: Foot-and-Mouth-Disease-Virus
GST: Glutathione S-transferase
LB: Luria Bertaini
SDS-PAGE: Sodium dodecyl sulphate–polyacrylamide gel electrophoresis
FPLC: Fast protein liquid chromatography
LC/MS–MS: Liquid chromatography–mass spectrometry
CD: Circular dichroism
ELISA: Enzyme-linked immunosorbent assay
HRP: Horseradish peroxidase
QELS: Quasi-elastic light scattering
DLS: Dynamic light scattering
TEM: Transmission electron microscopy
CTF: Contrast transfer function
