# Supplementary figures and images for: Oligomers of hepatitis A virus (HAV) capsid protein VP1 generated in a heterologous expression system
Source: Microb Cell Fact. 2022 Apr 7;21:53. doi: 10.1186/s12934-022-01780-x (PMC8991588; doi:10.1186/s12934-022-01780-x)

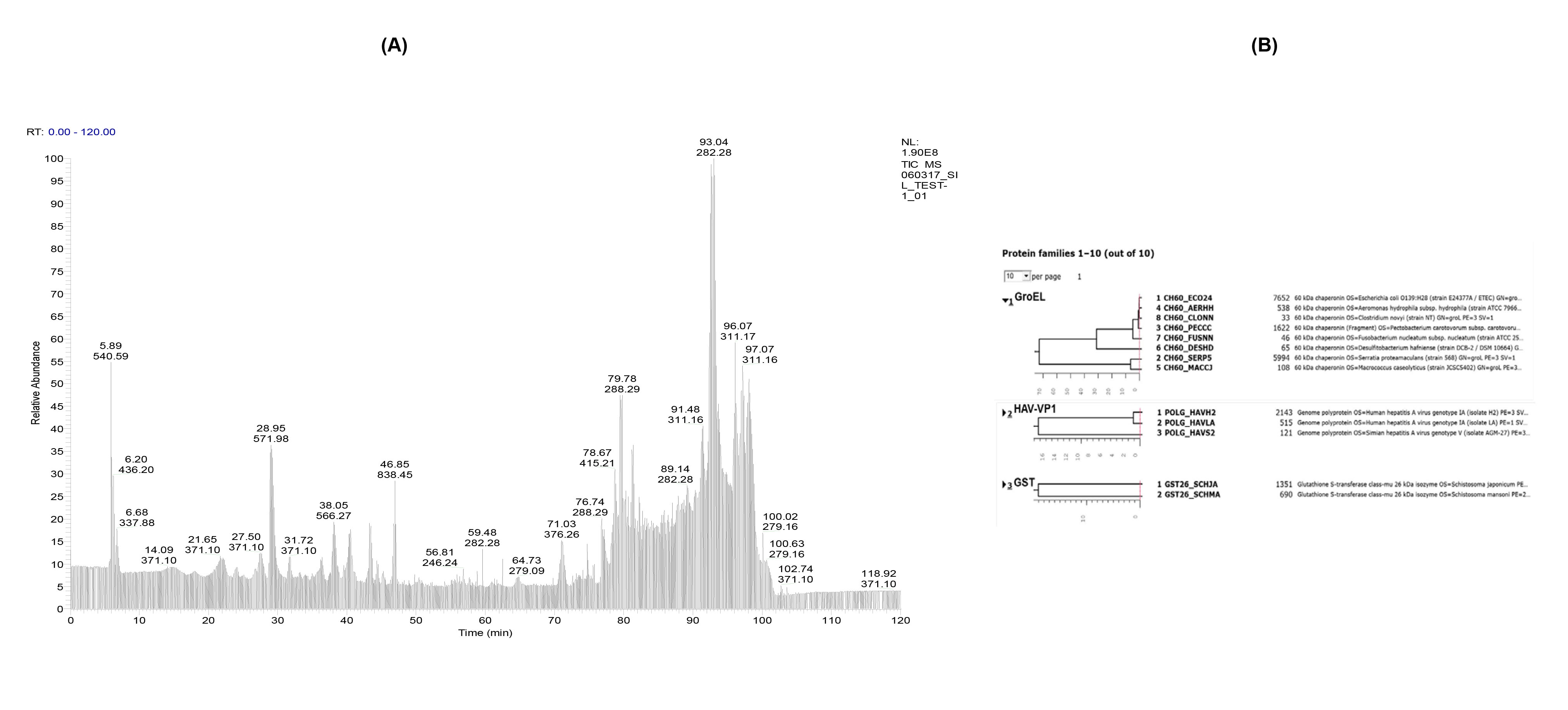

Supplement: Supplementary file 1 — Additional file 1: Figure S1. Association of His-GST-VP1 with bacterial chaperone GroEL determined by LC–MS/MS (A) LC–MS/MS analysis showing mass spectra of trypsin digested, purified protein (B) Peptide mass fingerprinting followed by database search confirmed the presence of GroEL, VP1 and GST. [file 12934_2022_1780_MOESM1_ESM.jpg]

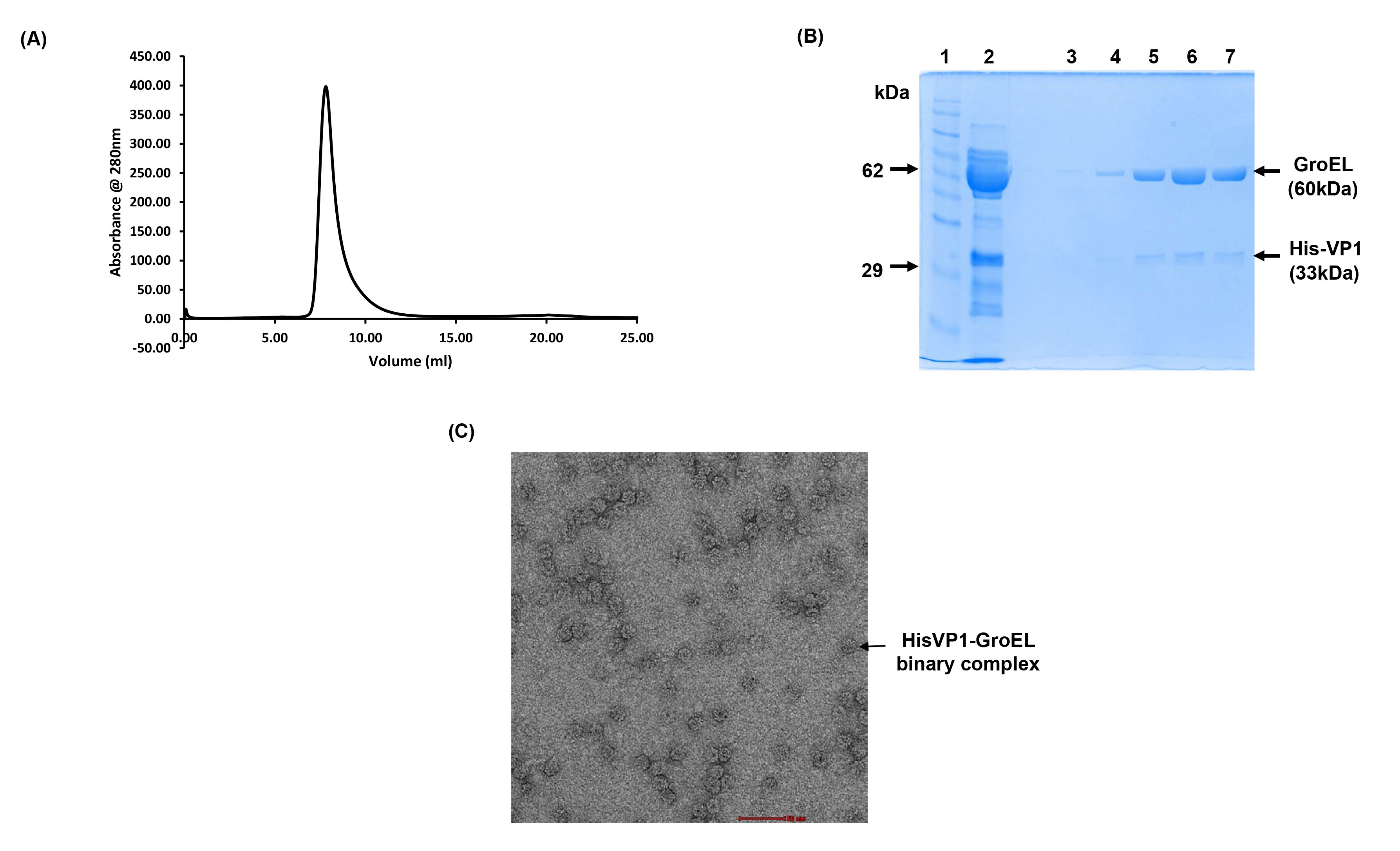

Supplement: Supplementary file 2 — Additional file 2: Figure S2. Expression and Purification of His-VP1 from E.coli strain Rosetta (DE3) pLysS cells (A) Elution profile of His-VP1 from a Superdex 200 (10/300) size exclusion column (B) Purified protein from size exclusion chromatography analyzed on 10% SDS-PAGE, showing the presence of both GroEL and His-VP1 at 60 kDa and 33 kDa respectively. Lane 1 represents the protein markers, lane 2 represents Ni–NTA purified fraction prior to SEC, lanes 3 to 7 represent the peak protein fraction from SEC (C) A complex of HisVP1-GroEL visualized by transmission electron microscopy. [file 12934_2022_1780_MOESM2_ESM.jpg]

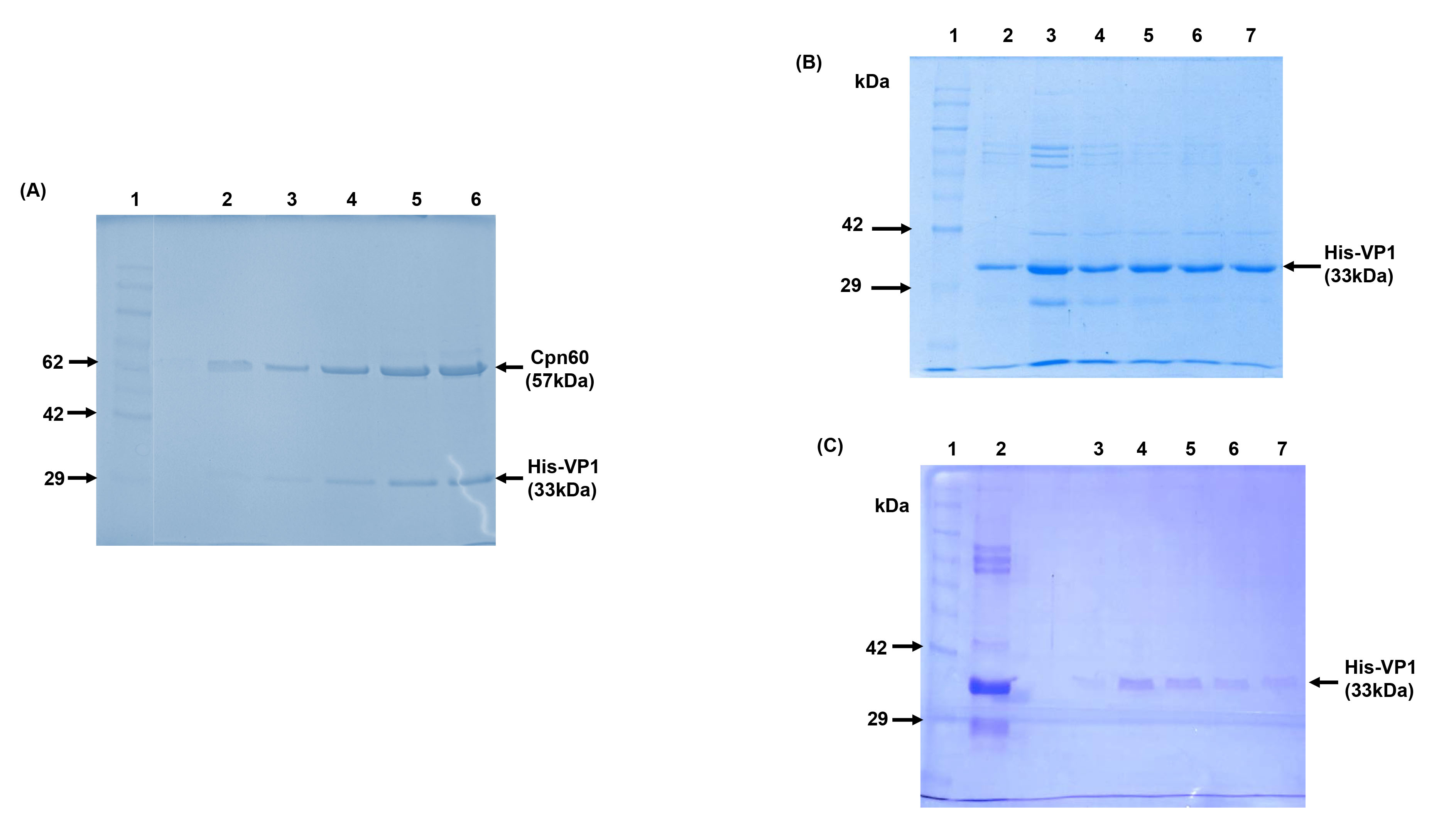

Supplement: Supplementary file 3 — Additional file 3: Figure S3. Expression and purification of His-VP1 from ArcticExpress (DE3) and BB1553 E.coli strains (A) Purified His-VP1 from ArcticExpress (DE3) cells analyzed on 8% SDS-PAGE. Size exclusion chromatography resulted in co-elution of Cpn60 and His-VP1. Lane 1 represents the protein markers, while lanes 2 to 6 represent the peak fraction from SEC. (B) Expression and purification of His-VP1 from E. coli strain BB1553 deficient in DnaK bacterial chaperone. Ni–NTA purified protein fractions analyzed on 10% SDS-PAGE. Lane 1 represents the protein markers, and lanes 2 to 7 represent protein fractions eluted with 350 mM imidazole. (C) SEC purified fractions of His-VP1 from E. coli strain BB1553 analyzed on 10% SDS-PAGE. Lane 1 represents the protein markers, lane 2 represents Ni–NTA purified fraction prior to SEC, lanes 3 to 7 represent the peak protein fraction from SEC. [file 12934_2022_1780_MOESM3_ESM.jpg]

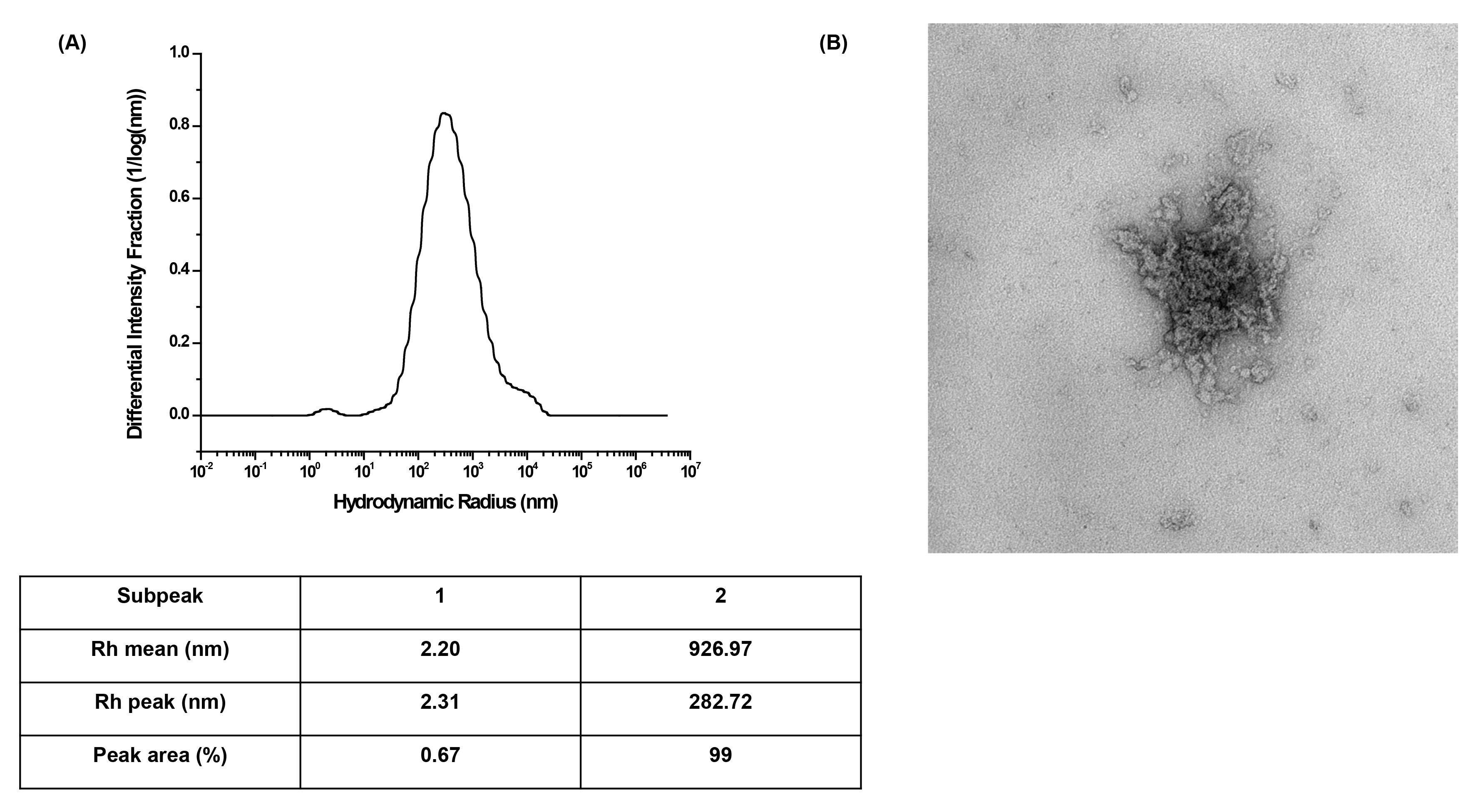

Supplement: Supplementary file 4 — Additional file 4: Figure S4. Size distribution and morphological characterization of VP1 purified from E. coli strain BB1553. (A) Dynamic Light Scattering (DLS) of purified VP1, with the Rhmean, Rhpeak and peak area percentage in a tabular form. (B) Presence of protein aggregate visualized by transmission electron microscopy. [file 12934_2022_1780_MOESM4_ESM.jpg]
